# A Clinical Lecture on a Case of Ulcerative Colitis

**Published:** 1909-05-22

**Authors:** J. Walter Carr

**Affiliations:** Physician to the Royal Free Hospital and to the Victoria Hospital for Children, Chelsea


### Hospital Clinics.

### A CLINICAL LECTURE ON A CASE OF ULCERATIVE COLITIS.

J. WALTER CARR, M.D., F.R.C.P.; Physician to the Royal Free Hospital and to the Victoria Hospital for Children, Chelsea.

The somewhat rare and at present mysterious disease which we term provisionally ulcerative colitis has lately attracted a good deal of attention, so that this affords a convenient opportunity of considering carefully the details of a fatal case which has recently been in the Royal Free Hospital, and which alike in its clinical manifestations, its course, its morbid appearances, and in the age of the patient, presents an exceedingly typical picture of the condition.

Mrs. W., æt. 33, was taken ill at the beginning of November 1908. As seems to be usually the case in ulcerative colitis, the patient's previous health had been perfectly good; she had been quite free both from diarrhœa and from constipation, the bowels having always acted regularly, and she had never lived abroad. There had been no illness among other inmates of the house. The onset of the disease was rather sudden, with diarrhœa six to eight times a day, and hæmorrhage from the bowel, bright red blood being passed in considerable quantity with each motion. Under treatment at home the hæmorrhage ceased, but as the diarrhœa continued and the patient was getting weaker, she was admitted to the Royal Free Hospital on December 14. The subsequent clinical history may be briefly summarised as one of persistent diarrhœa, occasional slight intestinal hæmorrhage, increasing weakness and wasting, and finally death on March 13, 1909, from exhaustion, due apparently to starvation, the emaciation being of the most extreme character, more marked even than in the majority of cases of malignant disease, and rivalling that met with in the worst examples of infantile marasmus.

Now let us consider the *Symptoms* more in detail. The most persistent was the diarrhœa. Probably from the commencement of her illness the patient never passed a normal motion; occasionally the stools were semi-solid, but usually quite liquid; sometimes they contained a little mucus and rarely some undigested food, but they were not specially offensive. Usually the bowels acted from three to six times within the 24 hours; there was often a little pain just previously, but not at any other time. At first the taking of food seemed to excite an action, but after a few weeks this ceased to occur. Tenesmus was sometimes complained of, but was never severe or persistent.

After the patient's admission to hospital the motions frequently contained small quantities of bright red blood, especially when the diarrhœa was more troublesome, but there was never any large hæmorrhage. There was no vomiting or hiccough. The abdomen was flat, but in the central part, especially just above the umbilicus, visible peristalsis was sometimes present; there was no tenderness, but some gurgling could be felt on pressure. The appetite was remarkably good throughout the greater part of the illness, and the food taken was probably satisfactorily digested. During the first few weeks the temperature was very irregular, rising at times to 102°. After the patient's admission to hospital its range was much lower; for several weeks it did not rise above 100°, and was at times quite normal for several consecutive days. Towards the end of life, however, it frequently rose to between 100° and 101°.

The blood was examined once (December 16), but merely showed the characters of an ordinary secondary anæmia: red blood corpuscles, 4,250,000; white, 12,600; an excess of small lymphocytes; no abnormal forms seen; hæmoglobin, 60 per cent.; colour index, .7.

At the *Autopsy* most extensive ulceration was found of the whole of the large intestine from the cæcum to within four inches of the anus, the ulcerated surface, except in the rectum, being much more extensive than the remaining mucous membrane. The ulcers were very irregular in shape, with slightly raised and much undermined edges, some of them communicating under ridges of intact mucous membrane. The peritoneal surface of the bowel, except for slight congestion, was normal, and there was no thickening of the wall of the gut. About a foot above the ileo-cæcal valve there was slight ulceration of the small intestine for two inches of its length, the ulcers being small and irregular in shape. The other organs were normal, except that the whole appearance was one of extreme inanition. Cultures from the ulcers grew bac. pyocyaneus and bac. coli; no amœbæ.

The *Diagnosis* in this case was not difficult, apart from the hesitancy one must always feel in giving a definite opinion in regard to a comparatively rare form of intestinal ulceration; but it is easy to realise that in less typical instances of the disease mistakes might easily be made. The essential thing is, of course, carefully to consider and exclude other possible causes of intestinal ulceration.

The considerable degree of pyrexia at the commencement of the illness raised the question of typhoid fever, but, apart from the great improbability of severe hæmorrhage occurring so early in typhoid, the other symptoms of that disease—the enlarged spleen, the abdominal distension, the rash, and the bronchitis—were all absent. That the distinction between the two diseases may, however, be very difficult is shown by the fact that last autumn a man died in the hospital from what was supposed to be a somewhat atypical attack of typhoid fever, ending in perforation, but at the autopsy the lesions were found to be those of ulcerative colitis, and were almost confined to the large bowel; it was in this that the perforation had occurred.

Tuberculous ulceration has to be thought of, but although common enough from secondary infection in cases of chronic pulmonary tuberculosis, it is very rarely met with in adult life as a primary lesion. Such primary ulceration occurs for the most part in children, at an age when true ulcerative colitis is very seldom seen. It may be noted also that severe hæmorrhage is a rare symptom in tuberculous ulceration of the bowel. As a further means of excluding the possibility of tubercle in this case, two injections of old tuberculin (.001 and .002 c.c. respectively) were given after the patient had been free from fever for some days; no rise of temperature followed.

The possibility of the symptoms being due to a new growth must always be considered, for in some recorded cases mistakes have arisen between the two conditions. A growth tends to produce obstruction rather than diarrhœa, but, as is well known, the irritation which it causes often gives rise to what the patient calls diarrhœa: observation of the stools soon shows that in reality very little actual fæcal matter is being passed. A rectal examination should never be omitted, although, of course, a neoplasm may be beyond the reach of the finger, but ulcers in colitis may sometimes actually be felt. In the present case the results of an ordinary rectal examination were negative, but valuable information was obtained by the sigmoidoscope, which showed that the upper part of the rectum and the lower end of the sigmoid flexure were studded with small ulcers. A swab of pus and blood taken directly from these ulcers was examined for tubercle bacilli, but none were found. It is well to bear in mind that the passage of the sigmoidoscope in these cases is not altogether free from risk, as the bowel wall may be greatly thinned and very rotten in places. At least one case has been recorded in which perforation has thus been brought about.

A duodenal ulcer is not likely to give rise to difficulty; it does not produce diarrhœa, and any blood passed will almost certainly be greatly altered in the course of its passage through the whole length of the bowel.

I have left to the last the diagnosis from dysentery, because at present it is doubtful whether we are in a position to draw any absolute distinction between the two conditions. Sir Patrick Manson has said very truly that "dysentery is not a disease, but merely a word standing for a group of symptoms indicative of an inflamed condition of the colon." The pathological appearances of dysentery and of ulcerative colitis seem to be identical. It may be noted that our patient, like most of those suffering from ulcerative colitis, and unlike those with ordinary dysentery, always had fæcal matter in the stools, and did not suffer much from tenesmus; but, after all, differences such as these are by no means essential, any more than is the fact that the patient had not been abroad, for dysentery is not confined to the tropics. It may be mentioned incidentally that we are equally in the dark as to the relations which the so-called "asylum dysentery" bears both to the tropical form and to ulcerative colitis. It may be that cases which we now call ulcerative colitis, and which seem to be entirely sporadic, bear the same relation to epidemic dysentery as do cases of simple posterior basal meningitis to epidemic cerebro-spinal fever. Possibly the final word on the question will be spoken by the bacteriologists, but at present they can scarcely be said to have uttered the first one.

In regard to *Prognosis*, there is no doubt that this is exceedingly grave, for of cases sufficiently severe to be definitely diagnosed probably at least 50 per cent. die, whilst of those which are lost sight of, it seems likely that a large proportion are not really cured, or at any rate relapse. The very extensive lesions commonly found at autopsies fully account for the extremely grave character of the disease.

*Treatment.*—This is most unsatisfactory, as may be inferred from what has already been said. It is obvious that a patient suffering from this disease should be kept at rest, warm, and should have hot applications to the abdomen in the event of there being any abdominal pain or discomfort. Some doubt may well exist as to the best method of feeding. In view of the extensive ulceration of the colon, it may be argued that the diet should be entirely fluid and unirritating, very much the same, in fact, as though the patient were suffering from typhoid fever. On the other hand, it often happens, as in the present case, that the tongue is clean, the appetite good, and digestion apparently performed quite normally. Under such circumstances, and assuming there is no considerable pyrexia, I believe it is better to give a fairly varied diet, including even some meat. Our present patient had such a mixed diet, and it never appeared in the slightest degree to increase either the diarrhœa or the hæmorrhage. She was also given soured milk, about two pints a day, for several weeks; but it did not produce any appreciable effect, and she greatly disliked it. It is strange that these patients should be able to take and apparently to digest large quantities of food, and yet, even in the absence of fever, should steadily lose weight, especially as there is little or no affection of the small bowel, in which presumably the greater part of digestion and absorption take place. Possibly, owing to some reflex influence, the food is unduly hurried along the small intestine.

Various drugs were tried by the mouth, including bismuth, tannigen, and paraffin, but the only one which was in any way useful was opium. For a long time it was given in the form of Dover's powder, ten grains every four hours. It never stopped the diarrhœa, but directly it was discontinued the motions became more numerous and more liquid. A remarkable feature was the way in which it was tolerated; a grain of opium every four hours never led to any contraction of the pupils or to drowsiness, and later on 15 minims of tinct. opii were given every two hours without such consequences resulting. Opium was also given per rectum, up to two grains at a time, but seemed to act better by the mouth. Possibly much of it escaped absorption, in the same way that the food apparently did, and this may explain the absence of its usual effects.

Locally, treatment was attempted by washing out the lower bowel with weak solutions of nitrate of silver (½ grain to the ounce), or a 2 per cent. solution of argyrol, followed in either case by a wash-out with a solution of boric acid (10 grains to the ounce) and common salt (3 grains to the ounce) in equal quantities. If any improvement at all followed, it was too slight to counter-balance the patient's great aversion to the process and the exhaustion to which it gave rise. The extensive lesions found at the autopsy throughout the whole length of the colon, equally in the upper as in the lower end, indicate that but little benefit is to be expected from treatment on these lines.

A few cases have been recorded in which great improvement has followed injections of coli vaccine, and accordingly on February 4 the patient was given an injection of 30 million bac. coli and bac. pyocyan. prepared from her stools, followed four days later by a second injection of 70 million bacilli. Unfortunately the results were apparently harmful rather than otherwise, as the temperature, which for some weeks before had not risen above 100°, afterwards rose for many days to a distinctly higher level, and at the same time the diarrhœa became rather worse.

Lastly, the question of operative interference was considered and suggested, but as both the patient and her relations were greatly opposed to it, the matter was not pressed. The subject is too large for discussion here, especially as the different aspects of the question will be found fully set forth in the Proceedings of the Royal Society of Medicine (Medical Section) for February and March 1909. Certainly an operation is not lightly to be undertaken, as the chances of success are very doubtful and the immediate risks in an advanced case by no means slight. Appendicostomy, followed by systematic washing out of the entire length of the large intestine, has several supporters. On theoretical grounds a right-sided colostomy presents greater chances of success, by diverting all fæcal matter from the ulcerated surface, but experience shows that any attempt to close the artificial anus is frequently followed by a return of the old symptoms.

In conclusion it must be acknowledged that at present our methods of treating the disease are altogether unsatisfactory. Probably not until we know more about its real nature and cause can we hope to attain better results.

